# Emerging Perspectives in Zinc Transporter Research in Prostate Cancer: An Updated Review

**DOI:** 10.3390/nu16132026

**Published:** 2024-06-26

**Authors:** Samantha Acevedo, María Fernanda Segovia, Erwin de la Fuente-Ortega

**Affiliations:** 1 Laboratorio Estrés Celular y Enfermedades Crónicas No Transmisibles, Departamento de Ciencias Biomédicas, Facultad de Medicina, Universidad Católica del Norte, Larrondo 1281, Coquimbo 1781421, Chile; 2 Centro de Investigación y Desarrollo Tecnológico en Algas y Otros Recursos Biológicos (CIDTA), Facultad de Ciencias del Mar, Universidad Católica del Norte, Coquimbo 1781421, Chile; 3 Núcleo de Investigación en Prevención y Tratamiento de Enfermedades Crónicas no Transmisibles (NiPTEC), Universidad Católica del Norte, Coquimbo 1781421, Chile

**Keywords:** zinc supplementation, zinc transporters, prostate cancer, signaling pathway, epithelial to mesenchymal transition

## Abstract

Dysregulation of zinc and zinc transporters families has been associated with the genesis and progression of prostate cancer. The prostate epithelium utilizes two types of zinc transporters, the ZIP (Zrt-, Irt-related Protein) and the ZnTs (Zinc Transporter), to transport zinc from the blood plasma to the gland lumen. ZIP transporters uptake zinc from extracellular space and organelle lumen, while ZnT transporters release zinc outside the cells or to organelle lumen. In prostate cancer, a commonly observed low zinc concentration in prostate tissue has been correlated with downregulations of certain ZIPs (e.g., ZIP1, ZIP2, ZIP3, ZIP14) and upregulations of specific ZnTs (e.g., ZnT1, ZnT9, ZnT10). These alterations may enable cancer cells to adapt to toxic high zinc levels. While zinc supplementation has been suggested as a potential therapy for this type of cancer, studies have yielded inconsistent results because some trials have indicated that zinc supplementation could exacerbate cancer risk. The reason for this discrepancy remains unclear, but given the high molecular and genetic variability present in prostate tumors, it is plausible that some zinc transporters—comprising 14 ZIP and 10 ZnT members—could be dysregulated in others patterns that promote cancer. From this perspective, this review highlights novel dysregulation, such as ZIP-Up/ZnT-Down, observed in prostate cancer cell lines for ZIP4, ZIP8, ZnT2, ZnT4, ZnT5, etc. Additionally, an in silico analysis of an available microarray from mouse models of prostate cancer (Nkx3.1;Pten) predicts similar dysregulation pattern for ZIP4, ZIP8, and ZnT2, which appear in early stages of prostate cancer progression. Furthermore, similar dysregulation patterns are supported by an in silico analysis of RNA-seq data from human cancer tumors available in cBioPortal. We discuss how these dysregulations of zinc transporters could impact zinc supplementation trials, particularly focusing on how the ZIP-Up/ZnT-Down dysregulation through various mechanisms might promote prostate cancer progression.

## 1. Introduction

Prostate cancer is the fifth leading cause of cancer-associated death and the second most common cancer in men [1]. This cancer predominantly arises from the peripheral prostate zone (80%), which contains the most abundant (>70%) and functional prostate glandular tissue [2]. In the normal prostate gland, the epithelial cell layer utilizes zinc for metabolic functions and synthesis of prostatic fluid, which is rich in citrate, specific proteases (PSA), and zinc [3]. To transport zinc from the blood plasma to the lumen of the gland, the prostate epithelium employs two types of zinc transporters, the ZIP (Zrt-, Irt-related Protein), which uptakes zinc from extracellular space (and from organelle lumen), and the ZnTs (Zinc Transporter), which release zinc outside the cells (or to organelle lumen) [4]. Several clinical and preclinical studies have demonstrated that prostate tumors exhibit zinc dysregulation, affecting zinc concentration levels and the expression of zinc transporters (see reviews [5,6,7,8]). Specifically, the downregulation of ZIP transporters (ZIP-Down) has frequently been correlated with low zinc levels in prostate tumors [5]. Interestingly, low zinc levels have been explored as a therapeutic target in this pathology [9,10,11,12,13]. However, results from preclinical and clinical trials of zinc supplementation have been contradictory. Some studies have suggested effectiveness against prostate cancer, while others have indicated that zinc intake may increase the risk of prostate cancer [14,15,16,17,18]. The reason for this discrepancy is unclear, but the high molecular and genetic variability in prostate tumors [19,20] suggests that these zinc transporters (14 ZIPs and 10 ZnTs) could be dysregulated in various patterns that respond differently to zinc supplementation. In this review, we present the current knowledge on the dysregulation of zinc transporters in prostate cancer. Additionally, we provide a novel in silico analysis of zinc transporter expression using data from a microarray of a transgenic mouse prostate cancer model (Nkx3.1;Pten) and human prostate adenocarcinoma mRNA-Seq (from pan-cancer project, cBioPortal), which support these different expression patterns. Moreover, we propose new potential pathological mechanisms involving these dysregulated zinc transporters in prostate cancer progression.

## 2. Zinc Homeostasis in the Normal Prostate Gland

**Figure 1:**
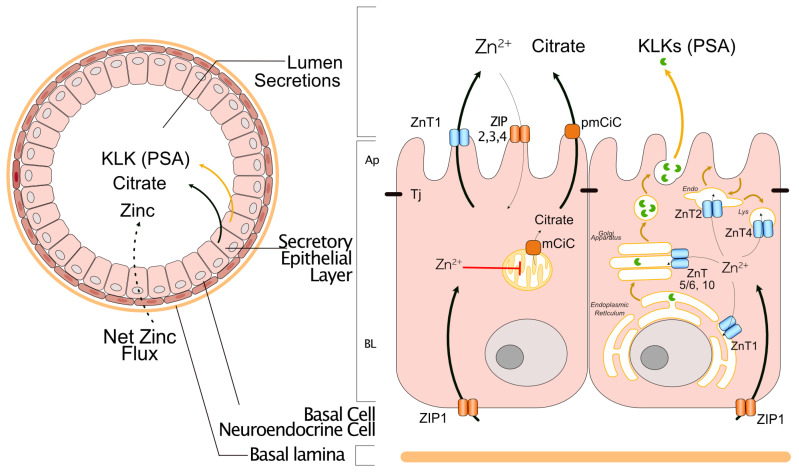
Zinc transporters mediate zinc homeostasis in the normal prostate gland. (**Left**), The secretory epithelium layer located in the lumen of the prostate acini synthesizes prostatic fluid, which is rich in citrate, kallikrein-related peptidase (KLK), such as PSA, and zinc. This epithelium expresses tight junctions (Tj), which prevent leakage of prostatic fluid components and maintain apical and basolateral polarity. (**Right**), Specific ZIPs and ZnTs distributed in the basolateral (ZIP1) and apical (ZnT1, ZIP2,3,4) surface membrane mediate the net zinc flux from the basolateral to the apical domain. Other potential zinc transporters are intracellular to regulate the zinc level inside the organelle lumen, including ZnT1 in ER, ZnT5/6 and 10 in Golgi apparatus, and ZnT2 and 4 in the endosomal-lysosomal system. Cytoplasmic zinc (Zn^2+^) can diffuse and inhibit the mitochondrial aconitase-2 enzyme, increasing citrate levels, which is secreted into the lumen through coordination of specific citrate transporters in mitochondria (mCiC) and plasma membrane CiC (pmCiC).

The prostate gland synthesizes prostatic fluid, which represents one-third to one-fifth of the ejaculate and contains specific proteases, citrate, and zinc (Figure 1, left). Typically, adult men (50–70 years old) have about 2–3 times more zinc in the prostate than young men (20 years old), which is distributed mainly on the lateral rather than the dorsal lobes [21]. The prostatic fluid concentrates 3–5 times more zinc than the prostate epithelium and stroma, which presents similar zinc levels to soft tissues like the liver, kidney, and skeletal muscle (15–30 mg/kg) [21]. In prostate epithelial cells, zinc plays a specific metabolic role, promoting the production of citrate by blocking the mitochondrial enzyme aconitase [22,23,24,25]. In addition, zinc in the organelle lumen of the biosynthetic pathway, including the endoplasmic reticulum (ER), Golgi apparatus, and endosomal-lysosomal system, can inhibit specific proteases called kallikrein-related peptidase enzyme (KLK3, and KLK2, 5 and 14) [26,27,28]. KLK3 is mainly known as the prostate-specific antigen (PSA), a biomarker of prostate cancer progression [29,30]. The high zinc concentration in the prostate fluid is required to prevent premature activation of KLK proteases. These proteases are only activated in the presence of zinc-binding proteins, known as semenogelins, which are released by seminal vesicles during ejaculation. This activation initiates the semen liquefaction process, which is necessary for sperm motility [27,28]. Consequently, the normal prostate must maintain adequate zinc homeostasis and regulate zinc levels in the organelle lumen, cytoplasm, and prostatic fluid. These controlled zinc levels ultimately govern the specific stages of sperm maturation.

Zinc homeostasis is regulated primarily by a family of 24 zinc transporters, which exhibit specific subcellular distribution and transport zinc in a directional manner (Figure 1, right) (the functions of these transporters were recently revised by [31]). The ZIP subfamily, comprising fourteen members in mammals, is encoded by solute carrier 39A genes (SLC39A1 to 14), while the ZnT subfamily, with ten members, is encoded by SLC30A1 to 10 genes [4,32]. Currently, only a subset of zinc transporters, including ZIP (ZIP1, ZIP2, ZIP3, ZIP4) and ZnT (ZnT1, ZnT2, ZnT4), have been identified and characterized in the normal prostate epithelium [33,34,35,36]. These transporters are polytopic transmembrane proteins that often form homodimers within the membrane. Each monomer contains a pore composed of conserved histidine and glutamate (or aspartate) residues that coordinate zinc [37,38]. ZIP functions as a uniporter, facilitating zinc movement from the extracellular space (or organelle lumen) into the cytosol, whereas ZnT acts as an antiporter (Zn^2+/^H^+^), transporting zinc from the cytosol to the extracellular space (or organelle lumen) [4,32]. The net zinc flux directed toward the gland’s lumen requires that zinc transporters be located specifically in the plasma membrane of prostate epithelial cells, either on the apical or basolateral plasma membrane. For instance, ZIP1 is located on the basolateral membrane to facilitate uptake of zinc from the bloodstream [3], while ZIP2 and ZIP3 are expressed apically, seemingly to retain zinc within the prostate epithelium by reabsorbing it from the prostate fluid [39,40]. Although the exact surface localization of ZIP4 in the prostate epithelium is unknown, it may be apically expressed in a manner similar to its distribution in the small intestinal epithelium [41,42]. ZnT1 is the only ZnT transporter expressed on the apical plasma membrane, responsible for mediating zinc efflux toward the acinus lumen [43]. The subcellular localization of several ZnTs has not been evaluated in the prostate epithelium, but studies in other epithelia suggest their intracellular presence. For example, ZnT4 is expressed in lysosomes and the Golgi apparatus [44,45], and ZnT2 has been found in secretory vesicles in mammary epithelial cells and Paneth cells, as well as in the endoplasmic reticulum, Golgi apparatus, and mitochondria [31,35,46,47,48]. Additionally, recent research has shown that ZnT3 and ZnT10 can be distributed in endosomes, potentially participating through zinc signaling in the modulation of tyrosine kinase receptors (TKR) cascade [49], and ZnT 5, ZnT6, and ZnT7 are expressed in exocytic pathway organelles, where they can play a regulatory role of several enzymes [44,45]. Further research is needed to understand the subcellular distribution of zinc transporters ZIP and ZnT in the prostate epithelium, which is crucial to elucidate their role in zinc homeostasis in normal or pathological states. 

Zinc homeostasis is also controlled by a coordinated expression between zinc transporter and cytoplasmic proteins known as metallothioneins (MT). In prostate tissue, there are three MT isoforms (MT1, MT2, and MT3) that can be regulated by metal levels, oxidative stress, and hormones [50,51]. For example, high cytosolic zinc levels can induce the expression of MT1 and MT2, which is coordinated with the upregulation of ZnT1 (and ZnT2). These changes help to reduce the cytosolic free zinc, increase the zinc efflux, and maintain zinc homeostasis [52,53]. Additionally, a coordinated expression between MT1 and zinc transporters (ZnT5, ZnT6, and ZnT7) can contribute to the regulation of zinc-dependent ectoenzymes (e.g., non-specific alkaline phosphatase) [54]. Consequently, these zinc transporter families, in coordination with MTs can mediate cellular zinc homeostasis, participate in zinc signaling, and modulate enzymatic activities.

## 3. Dysregulation of Zinc and Zinc Transporters Contributes to Pathological Mechanisms in Prostate Cancer

**Figure 2:**
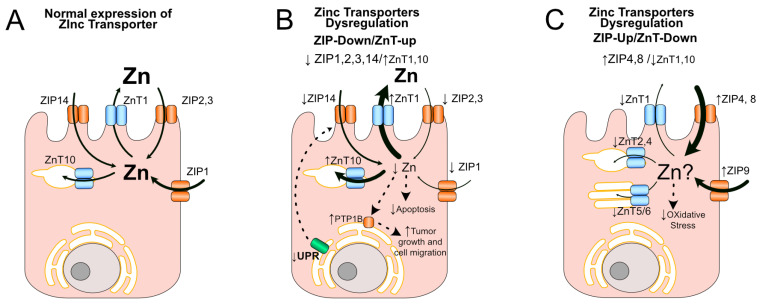
Patterns of zinc transporter dysregulation (ZIP and ZnT) in prostate cancer: (**A**) Subcellular distribution of zinc transporters in normal prostatic epithelial cells. ZnT1, ZIP2, ZIP3, and ZIP14 are localized apically, while ZIP1 is basolateral, and ZnT10 is situated within the endosomal system. Together, they transport zinc (solid arrows) and regulate cytosolic zinc levels (Zn). (**B**) Zinc transporter dysregulation commonly presented in prostate cancer tumors: Prostate cancer tumors present an expression pattern of ZIP-Down/ZnT-Up in zinc transporters, with the downregulation (↓) of ZIPs (ZIP1, ZIP2, ZIP3, and ZIP14) and the upregulation (↑) of ZnTs (ZnT1, ZnT2, ZnT9, and ZnT10) (see Table 1 and Table 2). This pattern contributes to low zinc levels (↓Zn), which could protect cells by several mechanisms, including decreasing the cytotoxicity zinc levels, decreasing apoptosis (dotted arrow), decreasing unfolding protein response (↓UPR) in the endoplasmic reticulum (ER), downregulation of ZIP14 through UPR signaling (dotted arrow), and increasing tumor growth and cell migration (dotted arrow). (**C**) Potential novel zinc transporter dysregulations observed in prostate cancer cell lines: Prostate cancer cell lines present a ZIP-Up/ZnT-Down expression pattern of zinc transporters, with an upregulation (↑) of ZIPs (ZIP4, ZIP8, ZIP9) and a downregulation (↓) of ZnTs (ZnT1, ZnT4, ZnT5/6). This pattern may affect cytosolic zinc levels (↑Zn), potentially protecting prostate cells from oxidative stress (dotted arrow) (additional mechanisms will be discussed in Section 6).

Prostate cancer undergoes metabolic reprogramming, in which the dysregulation of zinc and zinc transporters may play a central pathophysiological role [55,56]. Higher zinc levels are present in the normal prostate, benign prostate dysplasia (BPH), and the pre-malignant stages, the low- and high-grade prostatic intraepithelial neoplasia (LGPIN and HGPIN) [56]. In contrast, malignant (primary tumors) and metastatic stages frequently have low zinc levels [56]. Indeed, several studies have demonstrated dysregulation (downregulation or upregulation) of specific zinc transporters in prostate cancer (Table 1 and Table 2, and Figure 2). These investigations assessed changes in the relative expression of zinc transporters at the mRNA level using RT-qPCR, as well as protein levels using western blot or immunohistochemistry (IHC), utilizing a limited number of samples from human prostate cancer [39,57,58]. For example, downregulation has been shown for ZIP1, ZIP2, ZIP3, ZIP4, and ZIP14 (ZIP-Down) in prostate cancer tissues, which seemingly adapt malignant cells to the cytotoxic effects of high zinc levels [39,58,59] (Figure 2B). The downregulation of ZIP1 has been correlated with low zinc and low citrate levels, which are frequently observed in prostate cancer tissues [59,60]. The downregulation of ZIP1 may be mediated by two transcription factors, RREB-1 and AP2, which are also affected in prostate cancer [61,62,63]. Interestingly, these changes in ZIP1 expression (zinc and citrate levels) can be recapitulated in an adenocarcinoma of the mouse prostate (TRAMP) or in middle-aged rats fed with a zinc-deficient diet [64,65,66]. Other downregulated ZIPs in human prostate cancer tumors are ZIP2 and ZIP3 [39], changes that, at least for ZIP3, are dependent on the transcription factor AP2 [63]. Given that these two transporters are located at the apical membrane, a depletion of ZIP2 and ZIP3 in prostate cancer could reduce the uptake of luminal zinc by the prostate gland [39]. ZIP4 and ZIP14 also are downregulated in prostate cancer tissue by unknown mechanisms [40,58]. Perhaps the downregulation of ZIP14 could involve the attenuation of the unfolded protein response (UPR) (ATF4 and ATF6), a mechanism reported for ZIP14 in the liver [67]. Consistent with this idea, UPR is also attenuated in the Nkx3.1;Pten mouse model of prostate cancer [68]. Furthermore, it has been reported that ZIP14 could have an additional pathological mechanism involving zinc signaling and the activation of protein-tyrosine phosphatase 1B (PTP1B) [67], an enzyme that contributes to cell migration, invasion, and tumor promotion in prostate cancer [69]. In vitro studies using the prostate cancer cell line PC3 (versus control) have also shown the downregulation of ZIP5, ZIP7, ZIP8, ZIP11, ZIP12, and ZIP13 [70], which needs to be confirmed by other studies. Then, the downregulation of various ZIPs (ZIP-Down) has been observed in prostate cancer tumors and cells, which may protect the tumor against the cytotoxicity of high zinc levels. In contrast, other ZIPs can be upregulated (ZIP-Up) in prostate cancer tissue and prostate cancer cell lines (Figure 2C, Table 1). For example, it has been found that ZIP9 is upregulated in prostate cancer tissue [71]. It has a new function as a membrane androgen receptor (AR), which can signal both through G proteins and as an androgen-dependent zinc transporter [71,72,73]. Overexpression of ZIP9 in the PC3 cell can mediate androgen induction of apoptosis by a mechanism dependent on zinc signaling and G proteins [73]. Recently, Thomas et al. found that the natural product epicatechin can interact with ZIP9 as an agonist in mediating androgen-induced apoptosis of PC3 cells [74]. Testosterone can also induce migratory activity in a ZIP9-dependent way in LNCaP cells [75]. More studies are required to understand the complex relationship between androgens, ZIP9, and prostate cancer. On the other hand, studies in vitro in prostate cancer cells have shown the upregulation of several ZIPs, including ZIP4, ZIP6, and ZIP8, in different prostate cancer cell lines that represent different metastatic origins (LNCaP from the lymph node, PC3 from the bone, and DU145 from the brain), and tumorigenic potential (LNCaP, very low; PC3, moderated; and DU145, high) [40,70,76], but their pathological mechanisms are largely unknown in prostate cancer. Thus, in prostate cancer, ZIPs can be dysregulated in two different patterns of expression: downregulation (ZIP-Down) or upregulation (ZIP-Up), and both patterns could be associated with pathological mechanisms in prostate cancer progression.

Similarly to ZIP dysregulations, several studies have shown that ZnTs can be upregulated (ZnT-Up) or downregulated (ZnT-Down) in prostate cancer tumors or cells (Table 2, Figure 2B,C). For example, ZnT1, ZnT 9, and ZnT 10 are upregulated in prostate cancer tissue, and these changes in ZnT1 and ZnT10 have been correlated with low zinc levels in prostate tumors [57]. Similar to ZnT1, it is possible that these ZnTs protect the tumor by reducing the toxicity of high zinc levels. An additional pathological mechanism could be related to zinc signaling. In the PC12 cell line, which is derived from rat adrenal phaeochromocytoma, the ZnT10 distributed in the Golgi apparatus and endosomes, forming heterodimers with other ZnTs (e.g., ZnT3, ZnT4, or ZnT5), which can modulate the epithelial growth factor receptor (EGFR) activity through zinc signaling [77,78]. EGFR is frequently overexpressed in prostate cancer tumors associated with high-grade and advanced stages of this cancer [79]. In contrast, the ZnT-Down pattern has also been observed for ZnT4, 5, and 6 in prostate cancer tissue, and ZnT1, ZnT2, ZnT5, ZnT6, and ZnT7 in prostate cancer cell lines, including LNCaP, PC3, DU145, and others, such as C4-2B (from lymph node and moderated tumorigenic potential) [57] (Table 2), but their pathological mechanisms are largely unknown. Altogether, these studies show that specific zinc transporters could be dysregulated at least in two principal expression patterns in prostate cancer (Figure 2B,C): the pattern ZIP-Down/ZnT-Up, which is consistent with the low zinc levels observed in prostate tumors and potentially favors the adaptation of tumors to cytotoxic high zinc levels, and the pattern ZIP-Up/ZnT-Down, to date, characterized only for ZIP9 in prostate cancer tumors but observed also in several prostate cancer cells line with different origin and tumorigenic potential, suggesting a potential role of this pattern in prostate cancer progression. 

**Table 1:** Dysregulation of zinc transporter ZIP (SLC39A) in prostate cancer.

ZIPs (Gene Name)	Human Prostate Cancer	Prostate Cancer Cell Lines/Prostate Cancer Transgenic Mouse or Other Cancer	References
ZIP1 (*SLC39A1*)	DOWN, adenocarcinomatous glands negative in 15 of 22 samples, 68% ^a,b^.	DOWN, ZIP1 protein in TRAMP mouse model ^b,c^.	[3,80]
		DOWN in TMPRSS2-ERG.Pten and Hi-Myc	[64]
ZIP2 (*SLC9A2*)	DOWN, adenocarcinomatous glands negative in 21 of 24 samples, 87.5% ^b^	DOWN in TMPRSS2-ERG.Pten and Hi-Myc	[39,80]
ZIP3 (*Slc3SLC9A3*)	DOWN, adenocarcinomatous glands negative in 21 of 24 samples, 87.5% ^b^	DOWN in TMPRSS2-ERG.Pten and Hi-Myc	[39,80]
ZIP4 (*SLC39A4*)	DOWN in prostate cancer tissue sample (*n* = 14) compared with BPH (*n* = 20), samples collected in China Medical University ^a^. Downregulated in 3 of 4 adenocarcinoma samples, compared with BPH (*n* = 4) ^c^	UP, in prostate cancer cell lines 22RV1 and PC3, compared to DU145 ^a^.	[40]
		DOWN, in prostate cancer cell line DU145 ^c^	
ZIP5, 6, 7 (*SLC39A5*, *6 and 7*)	Unknown	Downregulated after incubation with zinc for 6 h	[70]
ZIP8 (*SLC39A8*)	N/A	UP, in prostate cancer cell lines, BPH-1, DU145, C4-2B, LNCaP and PC3 compared to normal prostate cancer cell lines (RWPE-1, RWPE-W99, CF-91, MLC8891) ^c^	[76]
ZIP9 (*SLC39A9*)	UP, in human prostate cancer tissues (acinar adenocarcinoma) paired (*n* = 4) ^a^.	N/A	[71,72,73]
ZIP10, 11, 12 and 13 (*SLC39A10*, *11*, *12* and *13*).	Unknown in prostate cancer tissue. They were upregulated in other cancer tissues, ZIP10 in human hepatocellular carcinoma compared to normal liver samples (*n* = 20) ^a^ and (*n*= 70 out of 95 HCC tissues) ^b^, samples collected in China, Guangdong Provincial People’s Hospital.	Downregulated in PC3 after incubation with zinc for 6 h	[70,81,82,83,84]
ZIP14 (*SLC39A14*)	DOWN, in prostate cancer (*n* = 150) compared with benign (*n* = 29) ^a^. Down, ZIP14 protein level (*n* = 98) compared with benign (*n* = 81) ^c^.	N/A	[85]

Abbreviations: BPH: benign prostatic hyperplasia; ^a^ mRNA relative levels; ^b^ Immunohistochemistry (IHC); ^c^ western blot. N/A: not applicable.

**Table 2:** Dysregulation of zinc transporter ZnT (SLC30A) in prostate cancer.

Zinc Transporters Gene/Protein	Human Prostate Cancer	Evidence from Prostate Cancer Cell lines/Prostate Cancer Transgenic Mouse or Other Cancer	References
ZnT1 (*SLC30A1*)	UP, in prostate tumor from European American (*n* = 13). Non-change, in prostate tumor from African American (*n* = 12) ^a^.	DOWN, in prostate cancer cell line * ^a^. UP, in TMPRSS2-ERG.Pten and Hi-Myc	[57,80]
ZnT2 (*SLC 30A2*)	No change or non-detected in human prostate cancer tissue samples (*n* = 25) ^a^.	DOWN, in human prostate cell line * except C4-2 ^a^. UP, in TMPRSS2-ERG.Pten and Hi-Myc	[57]
ZnT4 (*SLC30A4*)	No changes in prostate tumor from European American or African American ^a^. Upregulated in cancer grade group 3 ^a^.	UP, LNCaP, C4-2, and MDA PCa 2b are known to be derived from a metastatic site ^a^.	[57,86]
	DOWN, with weak staining in 112 (75%) and moderate or strong staining in only 37 (25%) cases.	DOWN, in most prostate cancer cell line DU145, 22Rν 1, PC3 ^a^.	
ZnT5 (*SLC30A5*)	DOWN, PCa tissue in both EA and AA populations (*n* total= 25) ^a^.	DOWN, in all tested prostate cancer cell lines * except MDA PCa 2b ^a^.	[57]
ZnT6 (*SLC30A6*)	DOWN, PCa tissue in both EA and AA populations (*n* total= 25) ^a^.	DOWN, in all human PCa cell lines * ^a^.	[57]
ZnT7 (*SLC30A7*)	Non-significative changes or non-detected in human prostate cancer tissue sample analyses (*n* = 25) ^a^.	DOWN, in all human PCa cell lines * ^a^. Knockout mice of SLC30A7 accelerate prostate tumor formation in TRAMP mice (65% in 2 weeks), suggesting insufficient SLC30A7 activity may contribute to PCa progression.	[57,87]
ZnT9 (*SLC30A9*)	UP, in combined analysis, samples from European and African Americans (*n* = 25) ^a^.	UP, in all human PCa cell lines * except in PC3 and 22Rv1 ^a^. UP, in TMPRSS2-ERG.Pten and Hi-Myc	[57,80]
ZnT10 (*SLC30A10*)	UP, in prostate tumors from European Americans (*n*= 13) and in prostate tumors from African Americans (*n* = 12) ^a^.	UP, in prostate cancer cell line 22Rν 1, LNCaP, C4-2, and MDA PCa 2b ^a^. UP, in TMPRSS2-ERG.Pten and Hi-Myc	[57,80]
		DOWN, in prostate cancer cell line DU145, PC3, E006AA-Par, and E006AA-HT ^a^.	

Abbreviations: ^a^ mRNA relative levels; * prostate cancer cell lines DU145, 22Rν 1, PC3, LNCaP, C4-2 and MDA PCa 2b.

## 4. Exploring Different Patterns of Expressions of Zinc Transporter by In Silico Analysis of Available mRNAs Databases

Different patterns of expression of zinc transporters could be due to the cellular and genetic heterogenicity observed in all cancers including prostate cancer [19,20,88,89,90]. Primary prostate cancer has shown alterations in oncogenes, including the androgen receptor (AR), the fusion of AR-response promotor and ETS (ERG), MYC, and PTEN, or mutations in tumor suppressors genes such as TP53 and NKX3.1 [2,89,91]. Typically, a unique combination of these genetic alterations is present in each tumor [92], and mutations are distributed with different frequencies correlating with tumor progression. For example, the mutation in the NKX3.1 gene is present in 5% of BPH, 20% of HGPIN, 34% of hormone-refractory prostate cancers, and 78% of metastases [93,94]. Genetically engineered mice with these human mutations have enabled the development of different mouse models of prostate cancer (e.g., TRAMP, Myc, Pten, Nkx3.1, etc.), which are widely used for testing the impacts on cancer progression, metastasis, and the effects of drugs for prostate cancer prevention and treatment [65,95,96]. Each mouse model can recapitulate certain aspects of prostate cancer [96]. For example, TRAMP generated by the expression of SV40 T antigen presents a phenotype characterized by androgen-independent tumors and neuroendocrine metastasis [97], while Nkx3.1;Pten mutant mice progress through all stage of prostate cancer to lymph node metastasis and castration resistance metastasis [95,98]. To date, the TRAMP prostate cancer mouse model has been utilized to explore the relationship between zinc homeostasis and prostate cancer [64,65,87]. In TRAMP mice, the ZIP1 transporter is downregulated [64], and the depletion of ZnT7 can accelerate prostate tumor formation [87]. The TRAMP mutant has some limitations because it serves as a model for a small population of patients, those who develop neuroendocrine metastases [99]. Recently, Prasad et al. found the downregulation of ZIP1, ZIP2, and ZIP3 and the upregulation of ZnT1, ZnT2, ZnT9, and ZnT10 in TMPRSS2-ERG-Pten and Hi-Myc mutant mice [80], suggesting that changes in zinc homeostasis could be an early event in prostate tumorigenesis. Further studies in other mouse models of prostate cancer are needed to explore how zinc transporters are dysregulated and how they may promote prostate cancer progression.

**Figure 3:**
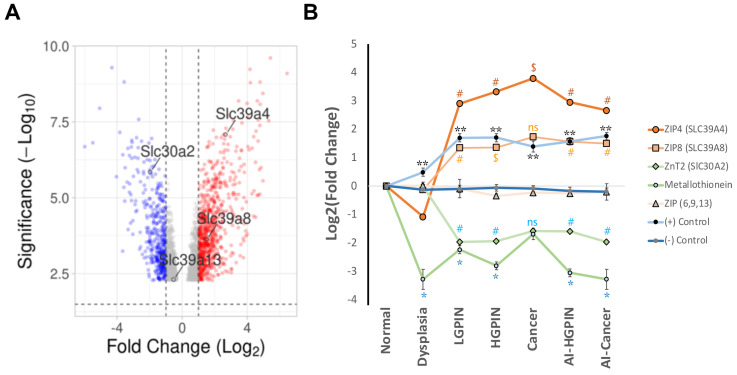
Dysregulation of zinc transporters in the Nkx3.1;Pten mice mutant. (**A**) Volcano plot of mRNA expression in cancer stage in the Nkx3.1;Pten mice mutant. The relative expressions of SLC30A and SLC39A were analyzed from the free microarray data of the Nkx3.1;Pten mice mutant, which are freely available in (GEO) NCBI (GSE11836) [98]. The volcano plot was made with the software VolcaNoseR [100] (https://huygens.science.uva.nl/VolcaNoseR) and shows the significant changes in mRNA (−Log_10_) versus fold change (Log_2_) (Dashed line: significance threshold 1.5, and fold change threshold +/− 1.0) Note that the prostate cancer stage of mice mutant showed significant upregulation of ZIP4 (SLC39A4) and ZIP8 (SLC39A8) and significant downregulation of ZnT2 (SLC30A2) (*p*-value < 0.05). (**B**) Fold change of zinc transporter mRNA through prostate cancer progression in the Nkx3.1;Pten mice mutant. The fold change of zinc transporters mRNAs was analyzed from the same database [98] in the different stages of prostate cancer, using the interactive software GEO2R [101] (https://www.ncbi.nlm.nih.gov/geo/geo2r/?acc=GSE11836). Note that from LGPIN to AI-cancer, significant upregulation of ZIP4 and ZIP8 mRNAs was shown, while ZnT2 was downregulated compared to control. The significant changes are shown as the *p*-value after adjustment (Adj. *p*-value) (# < 0.005 and $ < 0.02), statistically non-significant (ns)). Other zinc transporters mRNAs, such as ZIP6, ZIP9, and ZIP13, did not present significant changes in all stages compared with the normal stage (*t*-test). Metallothioneins (MT1 and MT2, average expression) were decreased significantly from dysplasia to AI cancer (except for cancer) (* *t*-test *p* < 0.05). As a positive control ((+) Control), it was used a set of 20 prostate cancer gene markers described by [98], which were upregulated significantly in all stages compared with the control (Average, standard error, ** *t*-test *p* < 0.003). As a negative control ((−) Control), it was used a set of 20 housekeeping genes described by [102]; their expression was compared with the control (average of Log_2_(foldchange) and standard error *t* test > 0.07)).

Fortunately, for the Nkx3.1;Pten mutant, there is an RNA microarray database available in the Gene Expression Omnibus at NCBI (GSE11836) [98], which we used to explore mRNA expression changes of zinc transporters by in silico analysis (Figure 3). The volcano plot showed significant changes, with the upregulation of ZIP4 and ZIP8 and the downregulation of ZnT2 in the cancer stage (Figure 3A). Interestingly, the changes of ZIP4 and ZIP8 commenced early in the progression (LGPIN) and persisted through HGPIN, AI-HGPIN, and Androgen-Independent (AI)cancer, while the changes of ZnT2 mRNA commenced at LGPIN and persisted until AI cancer (Figure 3B). Furthermore, no significant changes were observed in the expression of other ZIPs, such as ZIP6, ZIP9, and ZIP13, across the different stages of prostate cancer. On the other hand, metallothioneins (MT1 and MT2) were downregulated in this mouse model, from dysplasia to AI cancer, consistent with the low zinc levels observed frequently in prostate cancer tumors. Therefore, this analysis suggests the patterns of expression ZIP-Up/ZnT-Down (ZIP4,8 and ZnT2, respectively) could be present in specific prostate cancer mutants (Nkx3.1;Pten). This also predicts that changes in zinc homeostasis could appear at early stages of prostate cancer progression.

**Figure 4:**
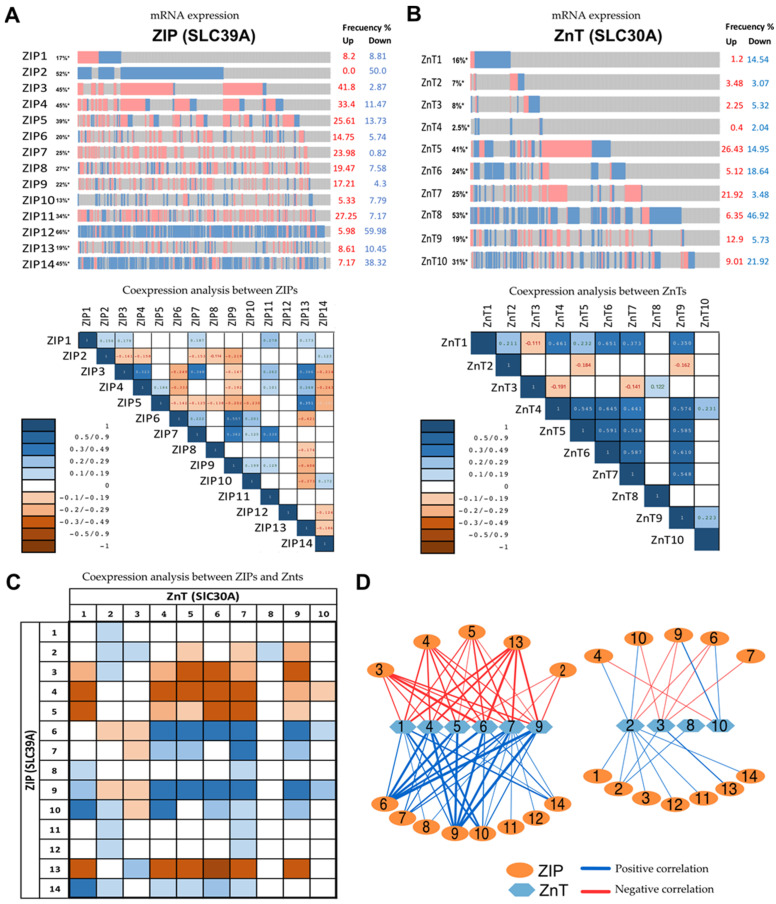
Human prostate adenocarcinoma presents a dysregulation of zinc transporters mRNA expression. (**A**) ZIPs transporter mRNA expression was analyzed from the free available database (cBioPortal) using the OncoPrint software (Z score threshold: 1.5) [103] (https://www.cbioportal.org). Each ZIP (1–14) shows the frequency of tumor (% from a total of 489 tumors) with upregulation (% of tumors in red) and downregulation (% of tumors in blue) in the right column. Below, the heatmap shows the significant Spearman’s correlation (q-value) between ZIP mRNA expression in adenocarcinoma. (**B**) ZnTs transporter mRNA expression was analyzed similarly to (**A**), showing theoncoprint, frequency of tumors, and the heatmap of Spearman’s correlation between ZnTs transporter mRNA. (**C**) Heatmap table of the coexpression analysis between ZnTs and ZIPs mRNAs shows the significant Spearman’s correlation (q-value < 0.05). (**D**) Interaction network represents the coexpression between ZIP and ZnT. The coexpression between ZIP and ZnT was graphic using Cytoscape software version 3.10.2 [105], using Spearman’s correlation data (q-value < 0.05) from C. Blue lines represent positive correlations, and red lines negative correlation between ZIP and ZnT mRNA in prostate adenocarcinoma samples.

To evaluate the expression patterns of zinc transporters in human prostate cancer tissue, an in silico analysis of RNAseq data was performed from human adenocarcinoma prostate cancer available through the pan-cancer project (cBioPortal, https://www.cbioportal.org) [103]. It utilized the transcriptomic RNAseq data from 489 samples from prostatic adenocarcinoma (Figure 4) [89]. The in silico analysis revealed that tumors frequently downregulated ZIP1, 2, and 14 (ZIP-Down pattern) and frequently upregulated ZIP9 (29.0% of the total samples), which aligns with previous analysis of mRNA expression in human prostate cancer [39,71,80,104]. Interestingly, human tumors also frequently upregulate ZIP4 and ZIP8 mRNA (33.4 and 19.47%, respectively), a result consistent with the expression observed in the Nkx3.1;Pten mutant mice model (Figure 3). Other ZIPs (SLC39A), which have not been studied previously in prostate cancer, such as ZIP10, 12, and 13, were principally downregulated, while ZIP11 was upregulated (Figure 4B). On the other hand, the analysis of ZnT mRNA (SLC30A) revealed that human tumors mainly downregulated ZnT4 and ZnT6 and upregulated ZnT9, which is consistent with previous studies (see Table 2). Additionally, using Spearman’s correlation, mRNA co-expression of zinc transporters was analyzed within the ZIPs, within the ZnTs, or between ZIP and ZnT in tumors (Figure 4C,D). The ZIPs formed two principal co-expression groups, the group ZIP3, ZIP 4, ZIP 5, and ZIP 13 (ZIP-3/13-group) and the group ZIP6, ZIP7, ZIP10, and ZIP 14 (ZIP-6/14-group), while ZnTs formed one principal co-expression group in prostate tumors ZnT1, ZnT2, ZnT4, ZnT5, ZnT6, and ZnT9 (ZnTs-1/9 group) (Figure 4C,D). Notably, the ZIP-3/13 group exhibited a negative correlation with some ZnTs, while the ZIP-6/14 group showed a positive correlation with some ZnTs (Figure 4D), suggesting the presence of different pattern expressions in ZIP-Down/ZnT-Up and ZIP-Up/ZnT-Down in human prostate tumors.

Altogether, these two in silico analyses (Figure 3 and Figure 4) are consistent with the dysregulation of specific zinc transporters in a particular pattern expression; the pattern ZIP-Down/Znt-Up is recapitulated by prostate cancer mouse model TRAMP, Myc, or TMPRSS2-ERG.Pten (e.g., ZIP1, ZIP2, ZIP3, and ZnT1, ZnT9, and ZnT10), and these patterns appear in the RNAseq analysis. The pattern ZIP-Up/ZnT-Down (e.g., ZIP4, ZIP8, and ZnT2) appears in the genetic context of Nkx3.1;Pten mice mutant, and in the RNAseq in silico analysis (e.g., ZIP4, ZIP 5, ZIP6, ZIP8, ZIP11 and ZnT4, ZnT5, ZnT6), suggesting that this dysregulation could be involved in a new mechanism of prostate cancer progression (Section 6); however, this different pattern could also impact the zinc supplementation assay of prostate cancer patients.

## 5. Could the Dysregulation of Zinc Transporters Affect Zinc Supplementation Treatments?

**Figure 5:**
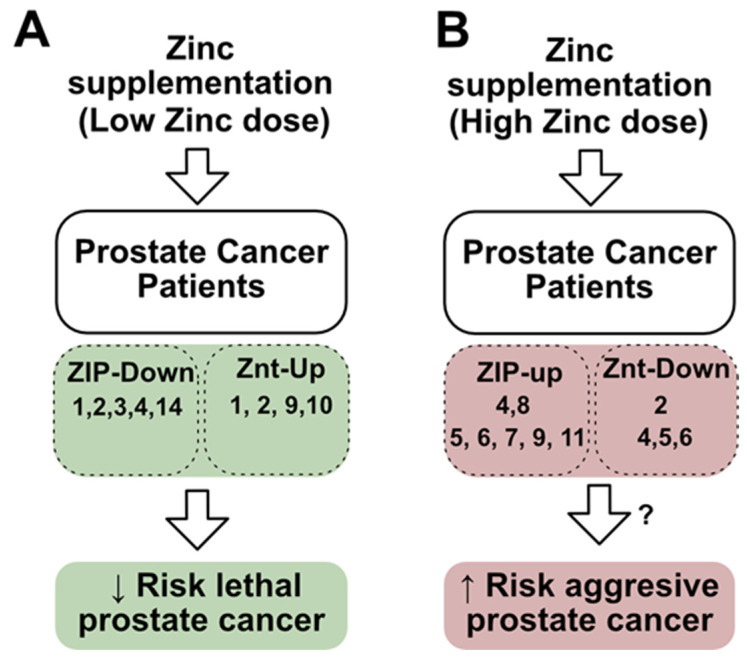
Hypothetical relationship between zinc supplementation, zinc transporter dysregulating, and effects in prostate cancer patients. (**A**) A low zinc dose of zinc supplementation has been correlated both with a decreased risk of lethal prostate cancer and with a ZIP-Down/ZnT-Up pattern expression (e.g., the humans ZIP1 to ZIP4 and ZIP14, and ZnT1, ZnT2, ZnT9 and ZnT10). (**B**) A high zinc dose of zinc supplementation has been correlated with an increased risk of aggressive prostate cancer and could be associated with a ZIP-Up/ZnT-Down pattern expression of zinc transporters (e.g., the mouse ZIP4 and ZIP8, and ZnT2, and humans ZIP5, ZIP6, ZIP7, ZIP9, and ZIP11, and ZnT4, ZnT5 and ZnT6). (↓: decrease, ↑: increase).

As previously mentioned, zinc supplementation has been hypothesized to have therapeutic potential against prostate cancer because a frequent biomarker in this pathology is zinc depletion in the prostate gland [106]. However, the effects of zinc supplementation are controversial, with results varying significantly according to mechanisms that are not well understood. Some studies have suggested that zinc supplementation may benefit prostate cancer patients by reducing the risk of lethal prostate cancer [107,108,109]. In contrast, recent studies, including a 30-year follow-up study and a multi-case control study in Spain, have indicated that zinc supplementation may increase the risk of lethal and aggressive prostate cancer [18,110]. Among the potential explanations for these discrepancies, the level of zinc supplementation (low or high doses) and genetic susceptibility to zinc have been suggested [18,110]. Zhang et. al. showed that a post-diagnostic low-dose zinc supplementation (1–24 mg/day) in nonmetastatic prostate cancer patients was associated with a lower risk of lethal prostate cancer [109], while high-dose zinc supplementation (>75 mg/day) may increase the risk of lethal and aggressive prostate cancer [18]. Interestingly, a Spanish population of prostate cancer patients receiving zinc supplementation (>10.53 mg/day) may be associated with deleterious effects in those patients with major polygenic risk scores [110], suggesting that a differential effect of zinc supplementation might depend on genetic susceptibility. According to this, we hypothesize that the dysregulation of zinc transporters could be part of the genetic susceptibility to zinc supplementation (Figure 5). As mentioned earlier, the pattern ZIP-Down/ZnT-Up (Figure 5A) could be correlated with the beneficial effects of zinc supplementation. However, the new pattern ZIP-Up/Znt-Down (Figure 5B), which is present in several prostate cancer cell lines and also observed in our in silico analysis of mRNA databases from prostate cancer mouse mutant Nkx31;Pten and human prostate tumors (RNAseq), suggests they could respond differently to zinc supplementation, such as by increasing the risk of aggressive of prostate cancer. Accordingly, the ZIP-Up/ZnT-Down pattern expression has been observed in several other cancers, promoting cancer progression by diverse mechanisms.

## 6. Potential Mechanisms of Pattern ZIP-Up/ZnT-Down in Prostate Cancer Progression 

**Figure 6:**
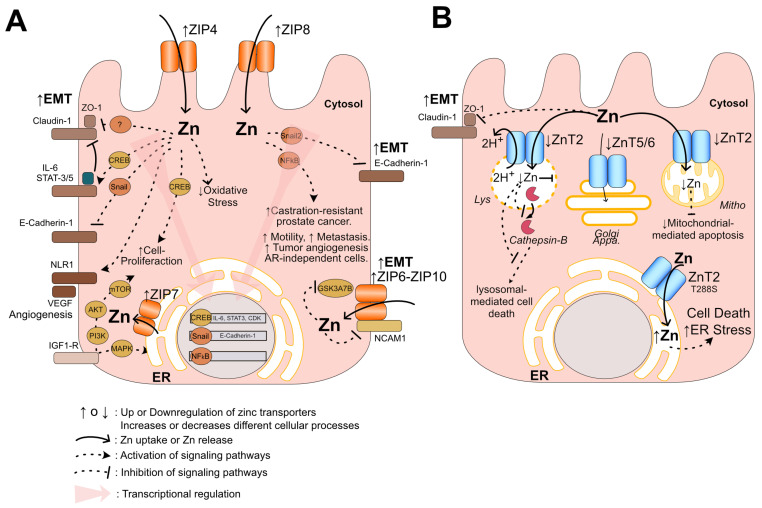
Potential pathological mechanism of pattern expression ZIP-Up/Zn-Down in prostate cancer progression. (**A**) Upregulation of ZIP4 and ZIP8 (↑) could mediate several pathological mechanisms, including the epithelial-to-mesenchymal transition (EMT), angiogenesis, cell proliferation, and antioxidant effect (oxidative stress protection). Zinc influx (solid arrows) by these transporters can exert signaling transduction pathways (dotted arrows) by activating transcriptional factors CREB, Snail, and NFkB. The transcription factor CREB can upregulate IL6 and STAT3, which inhibit tight-junction proteins, while Snail can downregulate E-cadherin or increase *N*-cadherin, increasing EMT (↑EMT) pathological process. In addition, CREB can upregulate CDK, which is involved in cell proliferation, and NFkB can mediate the increase of motility, metastasis, and angiogenesis of prostate tumors. Upregulation of ZIP7 (↑ZIP7) could stimulate cell proliferation through activation of the IGF1R cascade (dotted arrow) of kinases MAPK, PI3K/AKT, and mTOR. Upregulation of ZIP6/10 could modulate the EMT and the focal adhesion complex (GSK3A/B and NCAM1) through zinc signaling. (dotted arrows) (**B**) Potential mechanism mediated by downregulation of ZnT2, ZnT5/6. ZnT2 localizes in lysosomes, where its downregulation (↓) can inhibit lysosomal-mediated cell death by decreasing (↓) the membrane permeabilization and reducing the release of cathepsin-B from the lysosomal lumen. ZnT2 also localizes in mitochondria, where its downregulation can decrease (↓) mitochondrial-mediated apoptosis. ZnT2 also localizes at the endoplasmic reticulum (ER), and its downregulation can decrease the ER stress-mediated cell death mechanism. Downregulation (↓) of ZnT5/6 heterodimer localized in Golgi apparatus could increase (↑) EMT by zinc signaling.

Although there are several hypothetic mechanisms how zinc excess can increase the aggressiveness of prostate cancer, they are poorly understood, especially because they fail to explain how zinc can specifically affect prostatic epithelial cells. For instance, an in vitro study has shown that zinc can induce the enhancement of telomerase activity [111], which has been implicated in prostate cancer tumorigenesis and tumor progression [112]. Other studies have proposed that zinc could induce insulin-like growth factor I (IGF-1) [113,114], a hormone that has also been involved in prostate cancer progression [115]. Additional studies have suggested that zinc could indirectly promote prostate cancer by affecting the immune response [116]. The in silico analysis suggests that the novel pattern expression ZIP-Up/ZnT-Down could mediate other mechanisms. This section focuses on discussing the potential mechanisms of the ZIP-Up (ZIP4, ZIP6, and ZIP8) and ZnT-Down (ZnT2, ZnT5, and ZnT6) patterns (Figure 6), drawing from their known functions and pathological roles in other cancers.

Previous studies by Chen and coworkers using a limited number of prostate cancer samples from the hospital of China Medical University have shown the upregulation of ZIP4 in one-quarter of the cancer samples compared with BPH [117]. ZIP4 upregulation has been observed in several cancers, including pancreatic cancer [85,118], ovarian cancer [119], nasopharyngeal carcinoma [120], and hepatocellular carcinoma [121,122]. Its upregulation has been correlated with oncogenesis and cancer progression, and it has also been considered a novel diagnostic and prognostic marker for human pancreatic cancer [85,118]. In hepatocellular carcinoma, ZIP4 upregulation has been associated with tumor size, metastasis stage, and recurrence [123]. Additionally, ZIP4 was an independent predictor of shorter overall survival in hepatocellular carcinoma patients [123] and higher glioma grade [124]. These studies suggest that ZIP4 upregulation could favor cancer progression through several mechanisms, including antioxidant protection, cell proliferation, epithelial-to-mesenchymal transition (EMT), and angiogenesis (Figure 6A). In pancreatic cancer, ZIP4 overexpression can protect cells from apoptotic cell death induced by zinc deficiency [125], possibly by a mechanism involving the antioxidant effects. In addition, overexpression of ZIP4 in the human pancreatic cell line can activate cell proliferation and EMT by the transcriptional induction IL6 pathway, including IL-6, Cyclin D1, STAT-3 [126,127], and the CREB/microRNA-373 axis [128]. Interestingly, STAT3/5 is involved in prostate cancer progression by driving EMT programming that enhances cell migration, invasion, and metastases [129,130]. Furthermore, in a pancreatic cancer cell line, ZIP4 can facilitate the EMT by decreasing tight junction proteins, including ZO-1 and claudin-1 [131]. In lung cancer, ZIP4 can promote cell migration, invasion, and metastasis both in vitro and in vivo of lung metastasis models by activating the Snail-N-cadherin signaling axis [132]. The Snail pathway is part of different signaling transduction pathways (TGFβ, IGF, EGF) involved in EMT and is considered a master regulator of EMT in prostate cancer [133]. Conversely, depletion of ZIP4 by RNAi reduces cell proliferation, migration, and the invasion of pancreatic cancer cell lines [134] or reverses EMT transition, increasing epithelial marker E-cadherin and reducing the mesenchymal marker vimentin in human nasopharyngeal carcinoma cells [120], suggesting ZIP4 as a potential therapeutical target for various type of cancers. On the other hand, ZIP4 can facilitate angiogenesis by increasing the expression of the vascular endothelial growth factor (VEGF) and its receptor neuropilin-1 (NLR-1), which (both NLR-1 and VEGFR), in other studies, have been involved in the progression and metastasis of prostate cancer [135,136,137,138]. 

In the in silico analysis, ZIP6 and ZIP7 were upregulated and coexpressed with ZIP10 in several human prostate tumors (Figure 4A). Interestingly, it has been shown that both ZIP6 and ZIP10 can form respective homodimers at the plasma membrane where they participate in zinc uptake, which can induce EMT. In addition, in mammary gland epithelium cells, ZIP6 can form a heterodimer with ZIP10, which can trigger EMT and mitosis [139,140]. Furthermore, proteomic and bioinformatic analyses have proposed that the ZIP6-ZIP10 heterodimer can form a plasma membrane complex with other proteins, including GSK3A/B and NCAM1, which, in the presence of zinc, could modulate the cellular focal adhesion [31]. On the other hand, ZIP7 is distributed along the exocytic pathway, including the ER, where it controls the luminal zinc level by moving zinc from the ER lumen to the cytosol [141]. In the MCF-7 breast cancer cell line, it has been shown that ZIP7 can modulate the IGF1-R cascade through zinc signaling that acts on kinases involved in proliferation, including MAPK, PI3K/AKT, and mTOR [142]. The activity of ZIP7 could be coordinated with ZIP6-ZIP10, through zinc-dependent kinases and phosphatases, to modulate cytoplasmic zinc levels involved in proliferation processes [31]. Furthermore, overexpressed ZIP7 can reduce the ER stress pathway, decreasing the apoptosis mediated by CHOP [143,144].

ZIP8 is the other mRNA upregulated in the in silico analysis. It localizes at the plasma membrane, but also in several organelles, including the ER, Golgi apparatus, and endosomes, and mitochondria [145,146,147]. Recently, Geng and coworkers found that overexpression of ZIP8 in mouse fibroblast cells (MEF) and transgenic mice induced morphological cell changes, with re-organization of the actin filament, increased cell proliferation and migration associated with the high expression of transcription factors NF-κB and Snail2 [148]. These transcription factors have been implicated in the progression of prostate cancer and are potential therapeutical targets for treating prostate cancer [149,150]. The NF-κB is part of both canonical (e.g., TNFR and IL-1R) and non-canonical (e.g., BAFFR) pathways that can promote the initiation and progression of castration-resistant prostate cancer [117], and as we mentioned earlier, Snail is a master regulator of EMT in prostate cancer [133]. Thus, these studies suggest that the ZIP-Up pattern of ZIP4, ZIP6, ZIP7, and/or ZIP8 could participate in prostate cancer by promoting EMT, proliferation, migration, and decreased apoptosis involving zinc signaling pathways.

On another hand, ZnT-Down of ZnT2, ZnT5, and ZnT6 could play a crucial role in cancer progression by EMT and defects in cellular death (Figure 5B). ZnT2 has been mainly studied in mammary epithelium (MEC), where it plays a crucial role in secreting zinc into breast milk [151,152]. In MEC, ZnT2 has been localized in several intracellular compartments, including the endosomal-lysosomal system proximal to the apical membrane [153], mitochondria [154], and endoplasmic reticulum [155]. Knockout mice for ZnT2 exhibited irregular anatomy and function of the mammary gland, and several mutants have been identified in humans ZnT2 (SlC30A2), which has impaired zinc secretion into breast milk and promotes transient neonatal zinc deficiency in breastfed infants [47,151,152]. Using the proton-motive force, ZnT2 transporters operate as Zn^2+^/2H^+^ exchangers to mobilize zinc inside the organelle lumen [156]. This transporter can participate in both physiological and pathological cell death [154,157,158,159]. Physiologically, the mammary gland undergoes involution, a process triggered by TNF-α, which increases ZnT2 expression at lysosomes [157]. This mediates zinc influx into the lysosome lumen, triggering cell death through lysosomal-mediated cell death (LCD) [157]. In general, LCD occurs by lysosomal membrane permeabilization (LMP), releasing lysosomal enzymes such as cathepsins into the cytosol, which induce cell death [160]. ZnT2 expressed in mitochondria can also mediate a late mammary gland involution, affecting ATP production and triggering apoptosis [154,157]. Furthermore, a genetic variant of ZnT2 (T288S) is redistributed toward the endoplasmic reticulum (ER), initiating ER stress, oxidative stress, and tight junction permeability [155]. These studies suggest that an eventual downregulation of ZnT2 in prostate cancer may protect tumoral cells from cell death mediated by LCD and intrinsic apoptosis pathways. Additionally, ZnT2 dysregulation could potentially be involved in the EMT mechanism. Mammary glands from ZnT2-null mice and in vitro nanospheres depleted of ZnT2 (3D culture with HC11 cells) showed structural cell defects with smaller apical areas and defects in polarity formation of 3D culture [161]. Consistent with this alteration, Lee and coworkers found that depletion of ZnT2 inhibits the PAR complex signaling, a complex involved in apical/basolateral polarity and tight junction barrier formation [161]. ZnT2 depletion promotes cytosol zinc signaling that affects the PAR complex (e.g., AKT, Axn2, CDC42) through phosphatase PTEN [161]. Other ZnT-Down are ZnT5 and ZnT6, which are coexpressed in prostate cancer tumors (Figure 4B). ZnT5 and ZnT6 are expressed in the Golgi apparatus and form functional heterodimers [45]. They participate in the biosynthesis of cancer-promoting zinc-requiring ectoenzymes such as alkaline phosphatase (ALP), autotaxin (ATX), or metalloproteinase (MMP-9) [44], and glycoprotein [162]. Depletion of ZnT5-ZnT6 heterodimer (or ZnT7 homodimer) decreases the growth of pancreatic cancer MIA PaCa-2 cells [162]. In addition, these ZnTs mobilize zinc to the ER lumen, which is necessary to activate ERp44 [163], a chaperone previously described as a biomarker of prostate cancer [164]. However, it is unclear how the downregulation of ZnT5 or ZnT6 could promote prostate cancer; a potential mechanism could be controlling zinc signaling by increasing cytosolic zinc levels, as described for the downregulation of ZnT6 in acute myeloid leukemia [165]. Thus, the ZnT-Down could promote prostate cancer progression by diverse mechanisms including EMT and cell death defects, mediated by zinc signaling.

## 7. Summary and Concluding Remarks

Prostate cancer is a complex pathology involving environmental and genetic factors. Preclinical and clinical research have found dysregulation of both the essential micronutrient zinc and its specific zinc transporter in the prostate gland of patients. Although the potential therapeutic target is the low zinc level found in prostate cancer, clinical trials of zinc supplementation have yielded conflicting results, emphasizing the necessity for further research.

In prostate tumors, there is cellular and genetic heterogeneity that could promote a genetic susceptibility to zinc supplementation, by poorly understood mechanisms. In this review, we summarize the principal dysregulation of zinc transporters in prostate cancer cell lines, prostate cancer tissue, a mouse model of prostate cancer, and in silico analyses of the microarray from a prostate cancer mice model (Nkx3.1;Pten) and a human RNAseq database (cBioPortal).

There are different patterns of expressions of zinc transporters in prostate cancer tumors. One pattern is ZIP-Down/ZnT-Up, which has been correlated both with low zinc in prostate cancer and a positive response to zinc supplementation. From our in silico analysis and prostate cancer cell lines, we found a new pattern expression, ZIP-Up/ZnT-Down, which suggests zinc transporters could respond differently to zinc supplementation. 

This ZIP-Up/ZnT-Down pattern could promote prostate cancer through several mechanisms. For instance, the upregulation of ZIP4, ZIP6, ZIP7, or ZIP8 could promote EMT, angiogenesis, cell proliferation, cell death defects, and antioxidant effects, while the downregulation of ZnT2, ZnT5, and ZnT6 could decrease cellular death pathways (lysosomal-mediated cell death, mitochondrial-mediated apoptosis, and ER stress-mediated cell death) or increase cytoplasmic zinc levels that operate in malignant zinc signaling. Further research is necessary to establish whether the ZIP-Up/ZnT-Down pattern of expression is associated with susceptibility to zinc supplementation and the risk of prostate cancer. 

## Abbreviations

AI cancer	Androgen-independent cancer
AR	Androgen receptor
AKT	Akt serine/threonine kinase family
AP2	Adaptor protein complex 2
ALP	Alkaline phosphatase
ATF	Activating transcription factor
ATX	Autotaxin
Axn2	*Axis inhibitor protein 2*
BAFFR	B-cell activating factor receptor
BPH	Benign prostate dysplasia
CDC42	Cell division cycle 42 protein
CHOP	C/EBP homologous protein
CREB	cAMP responsive element binding protein
EGF	Epidermal growth factor
EGFR	Epidermal growth factor receptor
EMT	Epithelial-to-mesenchymal transition
ERG	ETS-related gene
ER	Endoplasmic reticulum
Erp44	Endoplasmic Reticulum Protein 44
ETS	Erythroblast transformation-specific
GEO2R	Gene expression omnibus
GSK3A	Glycogen Synthase Kinase 3 Alpha
HGPIN	High prostatic intraepithelial neoplasia
IHC	Inmunohistochemistry
IGF-1	Insulin-like growth factor 1
IGF1-R	Insulin-like growth factor 1-receptor
IL-1R	Interleukin-1 receptor
IL-6	Interleukin 6
KLK	Kallikrein-related peptidase
LCD	Lysosomal-mediated cell death
LMP	Lysosomal membrane permeabilization
LGPIN	Low prostatic intraepithelial neoplasia
MAPK	Mitogen-activated protein kinase
MEC	Mammary epithelium
MEF	Mouse fibroblast cells
MMP-9	Metalloproteinase
MT	Metallothioneins
MTF-1	Metal regulatory transcription factor 1
mTOR	Mechanistic Target of Rapamycin
MYC	*Myelocytomatosis oncogene*
NCAM1	Neural cell adhesion molecule 1
NCBI	National Center for Biotechnology Information
NF-kB	Nuclear factor kappa B
*NKX3.1*	NK3 homeobox 1
NLR-1	Receptor neuropilin-1
PAR	Partitioning defective protein
PI3K	Phosphatidylinositol 3-kinase
PSA	Prostate-specific antigen
PTEN	Phosphatase and tensin homolog
PTP1B	protein-tyrosine phosphatase 1B
RREB-1	Ras responsive element binding protein 1
SNAIL	Snail family transcriptional repressor 1
STAT-3	Signal transducer and activator of transcription 3
TGFb	Transforming growth factor-beta
TMPRSS2	Transmembrane protease serine 2 gene
TNF-α	Tumor necrosis factor-alpha
TNFR	Tumor necrosis factor receptor
TRAMP	Transgenic adenocarcinoma of the mouse prostate
TP53	Tumor protein p53
UPR	Unfolded protein response
VEGF	Vascular endothelial growth factor
ZIPs	Zn-regulated, Iron-regulated transporter-like proteins (Zn influx proteins)
ZnTs	Zinc efflux transporter proteins
ZO-1	Zonula occludens-1
